# Death from Hæmorrhage Consequent upon Lancing the Gums

**Published:** 1852-08

**Authors:** F. A. B. Bonney


From the London Lancet.

*Death from Hæmorrhage Consequent upon Lancing the Gums.* By F. A. B. Bonney.

Sir.—Your correspondent, Dr. Whitworth, who thinks his case of fatal hæmorrhage from lancing the gums to be unique,* will find a similar one communicated by Mr. Taynton to the late *Medical Gazette*, so far back as January, 1836, besides at least two others in the second volume of the Lancet for 1846.

Mr. Taynton,—after stating that his little patient, six months old, had the gums lanced on Sunday, and in spite of various styptics, including as in Dr. Whitworth's case, the actual cautery, died from the loss of blood on the Tuesday following,—proceeds as follows:

"Now, supposing such a case had occurred in a family of high rank, and the child had died, what a sensation would it not have caused! And how highly injurious might it not have proved to the surgeon's reputation! A similar case might happen again. Surely, then, it is important to know what mode of treatment would be likely to arrest the hæmorrhage; and I hope that some of your able correspondents will favor us with their opinion on the subject."

As I am not aware that this appeal to the opinion of medical men was ever responded to, though the conjecture that cases of the same distressing kind might happen again has been repeatedly realized, I beg to offer you my own ideas on the subject:

The first point to consider is, how the occurrence may be prevented. If, as in Mr. Taynton's case, the hæmorrhagic diathesis exists, no one knowing this would think of scarification. It may be advisable, therefore, in every instance where that operation is indicated, and the child is under a twelvemonth, to inquire whether that peculiar constitution exists in the family. Surely, it is better to put the question a thousand times in vain than lose the chance of avoiding that most painful of all a medical man's trials—the sight of a helpless patient dying through the very means employed for his relief.

Another most important rule is to make no long incision, but if several teeth are advancing on the same line of gum, to let the scarification be short and detached. In one of the cases I have met with in the Lancet, the gum-fleam had slipped from over the crown of the tooth backwards, and had separated the gum to some extent from the inner surface of the jaw-bone. This, of course, should be carefully avoided; and it will more easily be so if no long incision is attempted.

Though never so unfortunate as to meet with troublesome bleeding from any gums I myself have lanced, the symptom has occasionally come under my treatment, and I have as yet nearly always

* *Vide* June Lancet, p. 488.

succeeded in getting it under by pressure with the finger upon a suitable compress, saturated with a strong solution of nitrate of silver. In one case only, that of Captain V——, do I remember employing the caustic in substance. That gallant officer, on the 4th of November, 1846, had been to a dentist, who had made a horizontal incision over the roots of the left upper bicuspids, which had bled the whole two miles of his walk home, and by the time I arrived had produced considerable pallor and faintness, and filled the mouth with coagulum. After applying the compress for a quarter of an hour, with partial effect, I saw the jet of a small artery, and after touching it with a point of lunar caustic, had no further trouble. With infants, however, on account of the delicacy of their mucous membrane, and especially with those whom I suspected of the hæmorrhagic diathesis, I would carefully avoid the solid nitrate of silver, for fear of secondary bleeding on the separation of the slough.

Independently of local measures, it would seem prudent to give from time to time a few drops of tincture of ergot or other internal styptic, (perhaps the tincture of matico,) so long as hæmorrhage continued; and, last in mentioning, though first to be remembered, one ought always, on learning that a child's gums were bleeding unusually after scarification, to *attend immediately*, which appears not to have been done in all the cases reported.

